# Supplementary material for: MOF-mediated histone H4 Lysine 16 acetylation governs mitochondrial and ciliary functions by controlling gene promoters
Source: Nat Commun. 2023 Jul 21;14:4404. doi: 10.1038/s41467-023-40108-0 (PMC10362062; doi:10.1038/s41467-023-40108-0)
Supplement: Supplementary file 1 — Supplementary Information [file 41467_2023_40108_MOESM1_ESM.pdf]

Supplementary Information for

MOF-mediated Histone H4 Lysine 16 Acetylation Governs Mitochondrial and Ciliary Functions by Controlling Gene Promoters

Dongmei Wang[1,3], Haimin Li[1], Navdeep S Chandel[3,4], Yali Dou[5] and Rui Yi[1,2,3*]

Affiliations:

[1]Department of Pathology, Northwestern University Feinberg School of Medicine, Chicago, IL 60611, USA

[2]Department of Dermatology, Northwestern University Feinberg School of Medicine, Chicago, IL 60611, USA

[3]Robert H. Lurie Comprehensive Cancer Center, Northwestern University Feinberg School of Medicine, Chicago, IL 60611, USA

[4]Department of Medicine, Northwestern University Feinberg School of Medicine, Chicago, IL 60611, USA

[5]Department of Medicine, University of Southern California, Los Angeles, CA 90033, USA

*Corresponding author. Email: yir@northwestern.edu

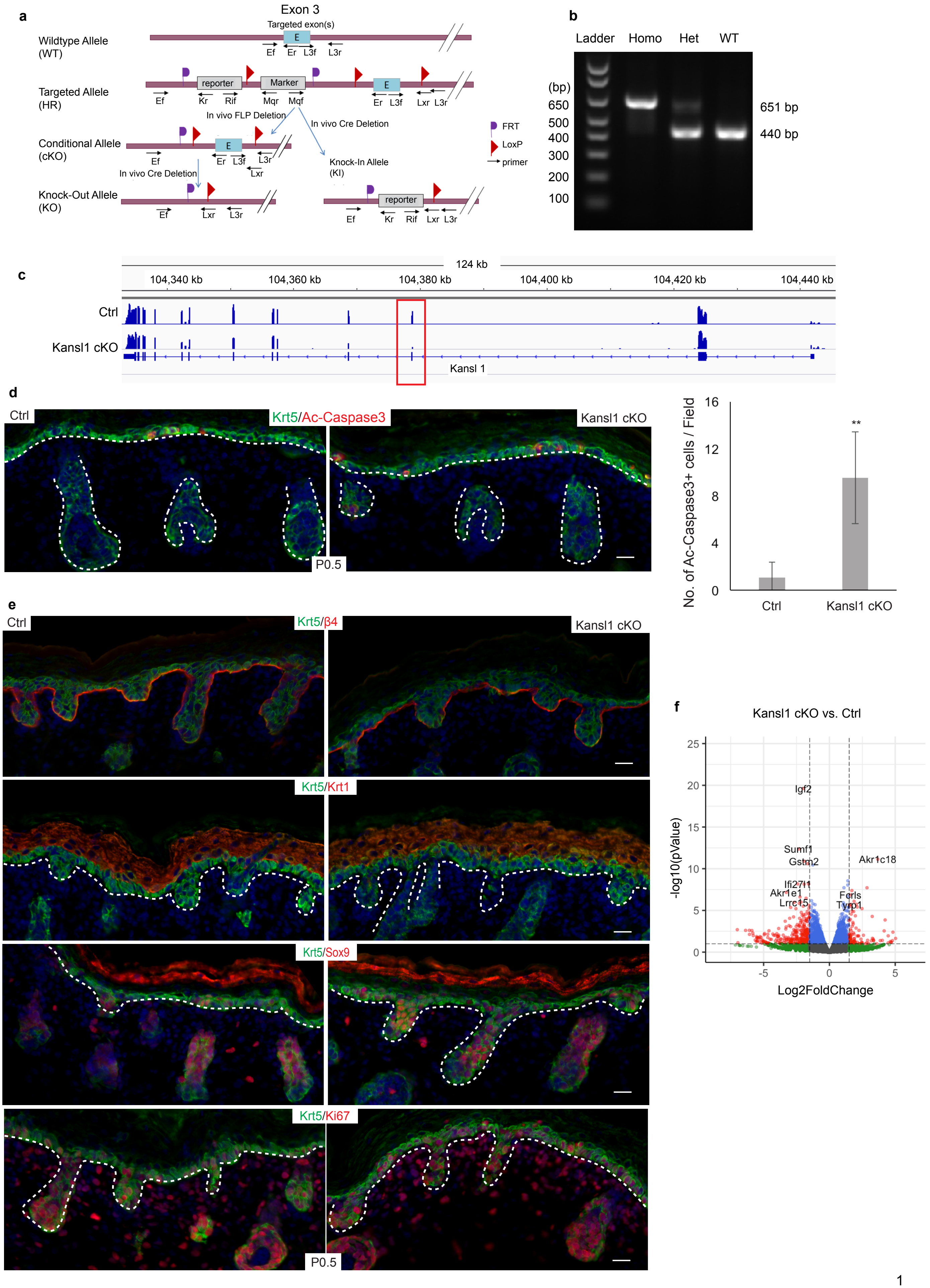
a
Exon 3
Targeted exon(s)
Wildtype Allele
(WT)
E
Ef
Er
L3f
L3r
Targeted Allele
(HR)
reporter
Marker
Kr
Rif
Mqr
Mqf
Lxr
In vivo FLP Deletion
In vivo Cre Deletion
Conditional Allele
(cKO)
Knock-In Allele
(KI)
Knock-Out Allele
(KO)
FRT
LoxP
primer
b
Ladder
Homo
Het
WT
(bp)
650
500
400
300
200
100
651 bp
440 bp
c
124 kb
104,340 kb
104,360 kb
104,380 kb
104,400 kb
104,420 kb
104,440 kb
Ctrl
Kansl1 cKO
Kansl 1
d
Ctrl
Krt5/Ac-Caspase3
Kansl1 cKO
P0.5
No. of Ac-Caspase3+ cells / Field
16
12
8
4
0
**
Ctrl
Kansl1 cKO
e
Ctrl
Krt5/β4
Kansl1 cKO
Krt5/Krt1
Krt5/Sox9
Krt5/Ki67
P0.5
f
Kansl1 cKO vs. Ctrl
-log10(pValue)
25
20
15
10
5
0
Igf2
Sumf1
Gstm2
Akr1c18
Ifi27l1
Akr1e1
Lrrc15
Ferls
Tyrp1
-5
0
5
Log2FoldChange

Supplementary Fig. 1 | Kansl1 knockout strategy and phenotypical analysis. a, Schematic illustration of the generation of Kansl1 conditional allele. **b**, Representative genotyping result from five sets of samples by genomic DNA PCR. Homo, homozygous, Kansl1 fl/fl; Het, heterozygous, Kansl fl/+; WT, wildtype, Kansl1 +/+. 651 bp is the conditional allele, 440 bp is the wildtype allele. **c**, RNAseq track showing deletion of exon3 in Kansl1 cKO. **d**, Increased apoptosis in P0.5 Kansl1 cKO shown by active-caspase 3 (Ac-Caspase3) staining. n = 21 fields for control, n = 32 fields for Kansl1 cKO from three pairs of samples. Data are represented as mean value +/- SEM. *P* value was calculated by unpaired two-sided Student's *t* test, **, *P* < 0.01. The exact *P* value is shown in Supplement Table 4. **e**, Immunofluorescence staining showing non-discernible difference between P0.5 control and Kansl1 cKO in basement membrane deposition (β4 integrin), differentiation and epidermal thickness (Krt1), hair follicle stem cell specification (Sox9) and proliferation (Ki67). Representative images from three pairs of samples. **f**, Volcano plot of differentially expressed genes in P0.5 Kansl1 cKO compared with control. Red dots represent genes showing absolute fold change > 1.5 and FDR < 0.01. Ctrl, control. White dashed lines indicate epidermal-dermal boundary (**d** and **e**). Scale bar, 20 μm (**d** and **e**). Source data are provided as a Source Data file.

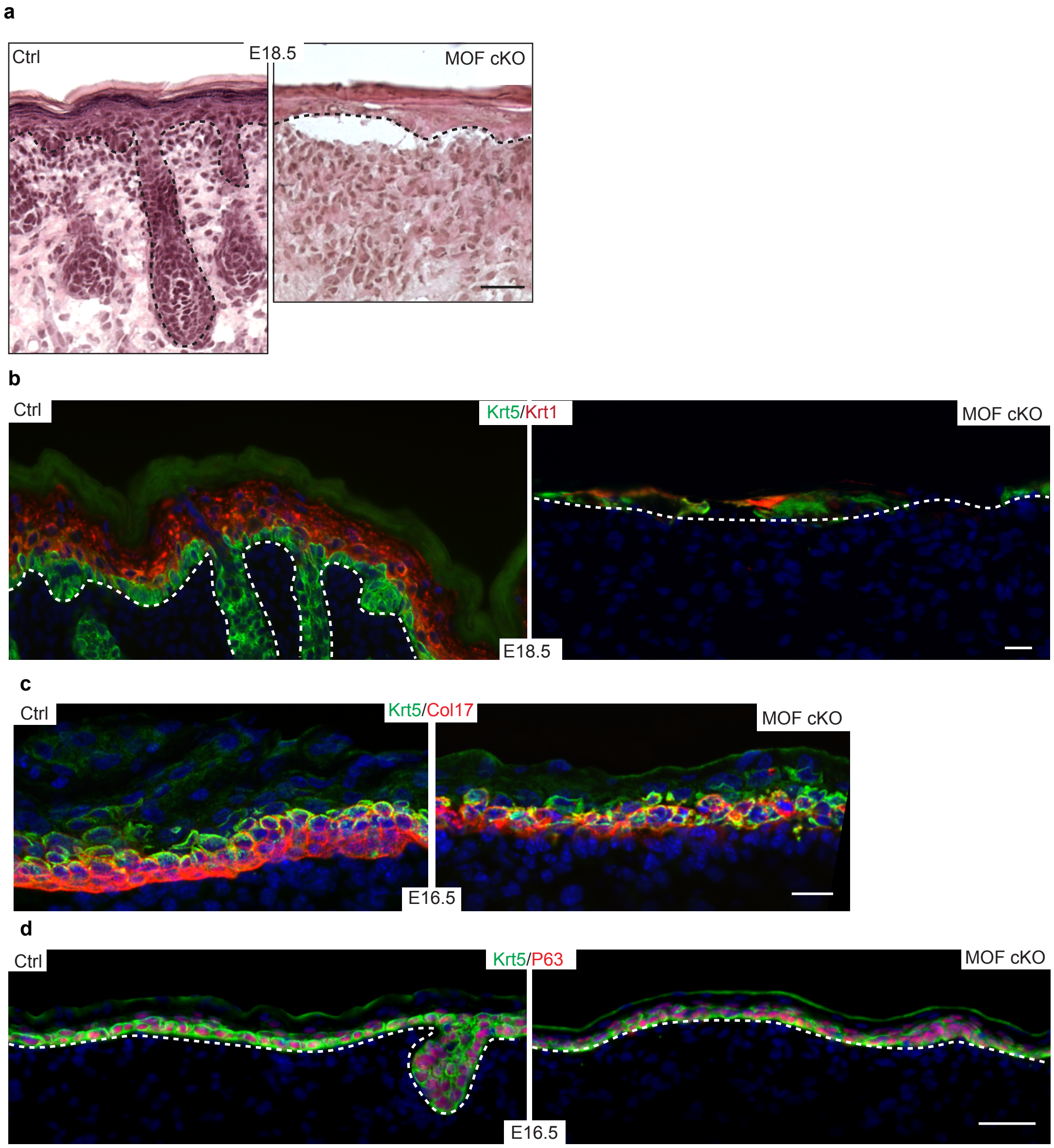

Supplementary Fig. 2 | Phenotypical analysis of MOF cKO. a, HE staining showing detachment of epidermis from dermis in MOF cKO at E18.5. Representative images from three pairs of samples. **b**, Loss of basal progenitor cells as indicated by Krt5 staining in E18.5 MOF cKO. Representative images from three pairs of samples. **c**, Compromised basement membrane in MOF cKO as shown by collagen XVII (Col17) staining. Representative images from three pairs of samples. **d**, Confirmation of epithelial fate specification in MOF cKO by epithelial master transcription factor P63 staining. Representative images from three pairs of samples. Scale bar, 50 μm (**a**), 20 μm (**b**-**d**).

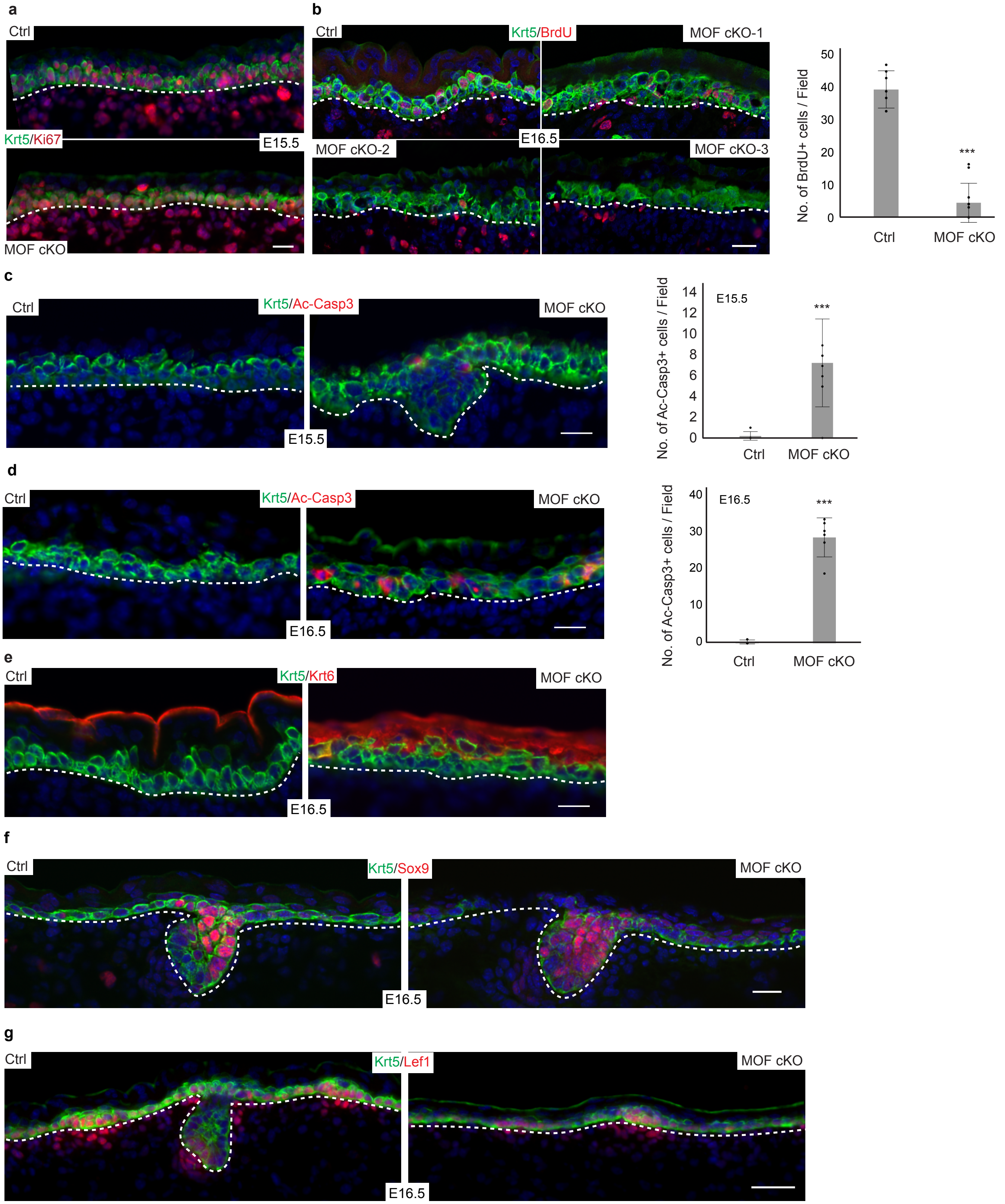
a
Ctrl
Krt5/Ki67
E15.5
MOF cKO
b
Ctrl
Krt5/BrdU
MOF cKO-1
E16.5
MOF cKO-2
MOF cKO-3
No. of BrdU+ cells / Field
50
40
30
20
10
0
***
Ctrl
MOF cKO
c
Ctrl
Krt5/Ac-Casp3
MOF cKO
E15.5
No. of Ac-Casp3+ cells / Field
14
12
10
8
6
4
2
0
E15.5
***
Ctrl
MOF cKO
d
Ctrl
Krt5/Ac-Casp3
MOF cKO
E16.5
No. of Ac-Casp3+ cells / Field
40
30
20
10
0
E16.5
***
Ctrl
MOF cKO
e
Ctrl
Krt5/Krt6
MOF cKO
E16.5
f
Ctrl
Krt5/Sox9
MOF cKO
E16.5
g
Ctrl
Krt5/Lef1
MOF cKO
E16.5

Supplementary Fig. 3 | More phenotypical analysis of MOF cKO. a, No significant change of cell cycle status in E15.5 MOF cKO as indicated by Ki67 staining. Representative images from three pairs of samples. **b**, Proliferation examination by BrdU staining in E16.5 samples. n = 7 fields for control, n = 11 fields for MOF cKO from three pairs of samples. Data are represented as mean value +/- SEM. **c**, Mild apoptosis was detected by active-Caspase 3 (Ac-Casp3) staining in E15.5 MOF cKO. n = 13 fields for each genotype from three pairs of samples. Data are represented as mean value +/- SEM. **d**, Extensive apoptosis was detected in E16.5 MOF cKO. n = 6 fields for each genotype from three pairs of samples. Data are represented as mean value +/- SEM. **e**, Stress response indicated by Krt6 staining in the epidermal differentiated layer in E16.5 MOF cKO. Representative images from three pairs of samples. **f**, Specification of hair follicle stem cells in the few hair follicles detected in E16.5 MOF cKO as shown by Sox9 staining. Representative images from three pairs of samples. **g**, Hair follicle specification in MOF cKO as demonstrated by Lef1 staining. Representative images from three pairs of samples. Ctrl, control. White dashed lines indicate epidermal-dermal boundary (**a**-**g**), *P* values were calculated by unpaired two-sided Student's *t* test (**b**, **c** and **d**), ***, $P < 0.001$. The exact *P* values are shown in Supplement Table 4. Scale bar, 20 μm (**a**-**g**). Source data are provided as a Source Data file.

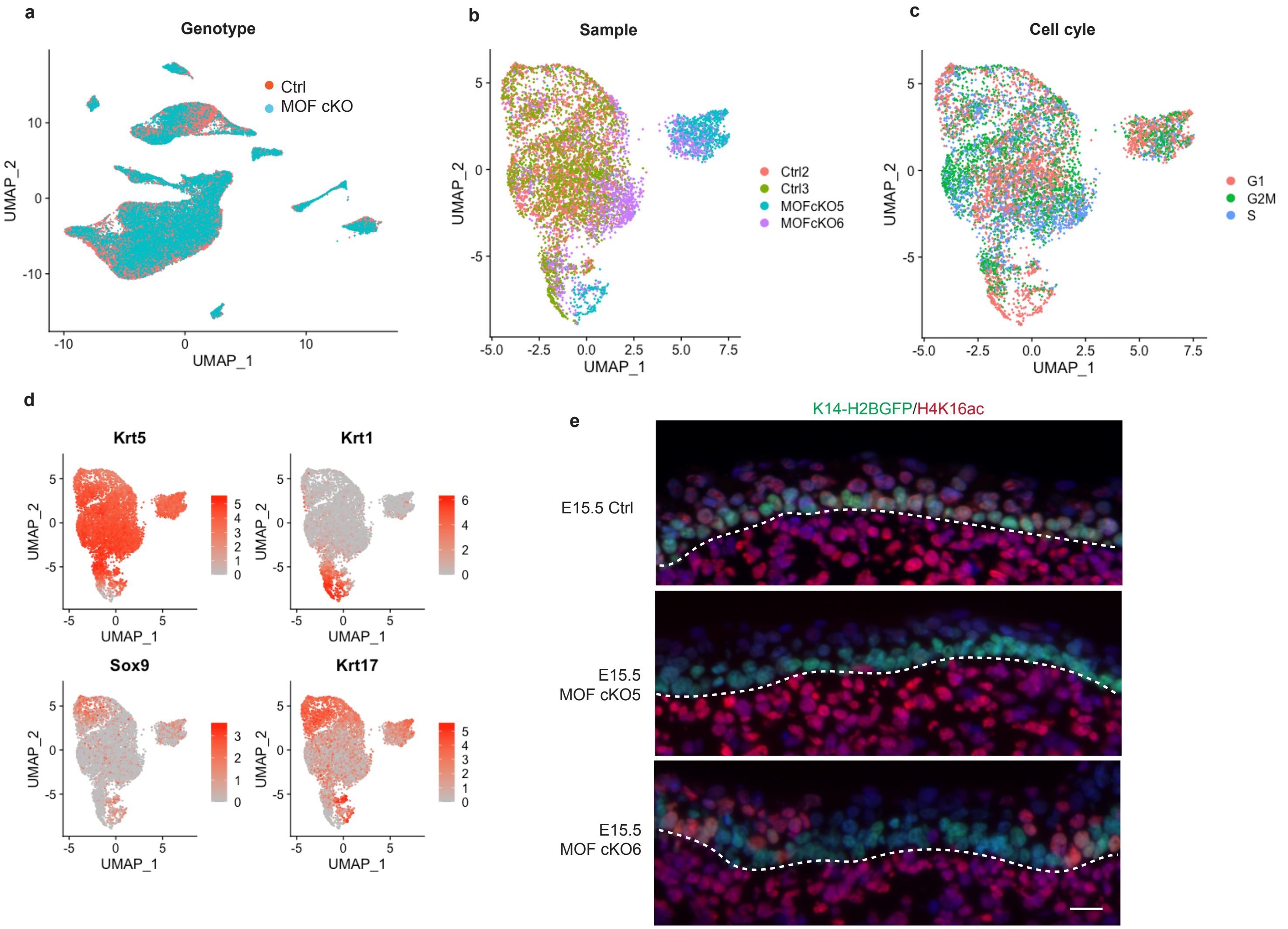

Supplementary Fig. 4 | E15.5 single cell RNAseq analysis and sample characterization. a, UMAP clustering of all the cells detected in E15.5 scRNAseq, colored by genotype. **b**, UMAP clustering of epithelial cells, colored by sample. **c**, UMAP clustering of epithelial cells, colored by cell cycle status. **d**, Feature plot of genes that were used for identifying epithelial clusters. Krt5 marks basal progenitor cells; Krt1 marks suprabasal cells; Sox9 mark hair germ cells; Krt17 marks hair germ cells in control and stress response cells in MOF cKO. **e**, H4K16ac staining for control and two E15.5 MOF cKO (MOF cKO5 and MOF cKO6) used in scRNAseq to show complete deletion of MOF in MOF cKO5 and mosaic deletion of MOF in MOF cKO6. Ctrl, control. White dashed lines indicate epidermal-dermal boundary, Scale bar, 20 μm.

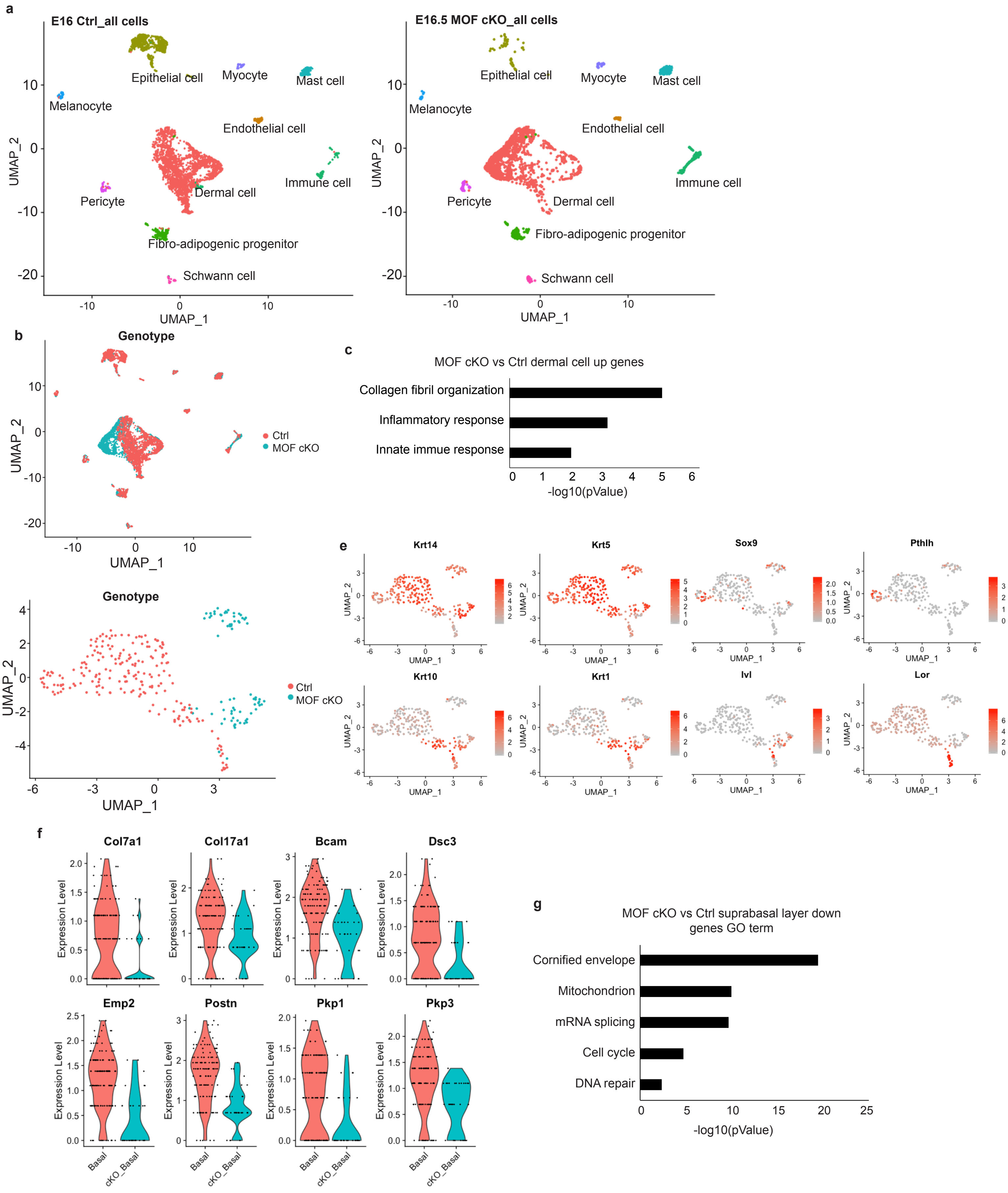
a
E16 Ctrl_all cells
E16.5 MOF cKO_all cells
Epithelial cell
Myocyte
Mast cell
Melanocyte
Endothelial cell
Immune cell
Pericyte
Dermal cell
Fibro-adipogenic progenitor
Schwann cell
UMAP_1
UMAP_2
b
Genotype
Ctrl
MOF cKO
c
MOF cKO vs Ctrl dermal cell up genes
Collagen fibril organization
Inflammatory response
Innate immue response
-log10(pValue)
d
Genotype
Ctrl
MOF cKO
e
Krt14
Krt5
Sox9
Pthlh
Krt10
Krt1
Ivl
Lor
f
Col7a1
Col17a1
Bcam
Dsc3
Emp2
Postn
Pkp1
Pkp3
Expression Level
Basal
cKO_Basal
g
MOF cKO vs Ctrl suprabasal layer down genes GO term
Cornified envelope
Mitochondrion
mRNA splicing
Cell cycle
DNA repair
-log10(pValue)

Supplementary Fig. 5 | E16.5 single cell RNAseq analysis. a, Ten distinct cell types were detected in E16.5 control and MOF cKO dorsal skin samples by scRNAseq. **b**, UMAP clustering of all the cells detected in E16.5 scRNAseq, colored by genotype. **c**, Gene ontology (GO) terms of up-regulated genes in E16.5 MOF cKO dermal cells. **d**, UMAP clustering of epithelial cells, colored by genotype. **e**, Feature plot of genes that were used for identifying each epithelial clusters. Krt14 and Krt5 mark basal progenitor cells; Sox9 and Pthlh mark hair follicle cells; Krt1 and Krt10 mark spinous layer cells; Ivl and Lor mark granular layer cells. **f**, Example of downregulated basement membrane and extracellular matrix genes detected in E16.5 scRNAseq. **g**, GO terms of downregulated genes in E16.5 MOF cKO *vs.* control suprabasal comparison.

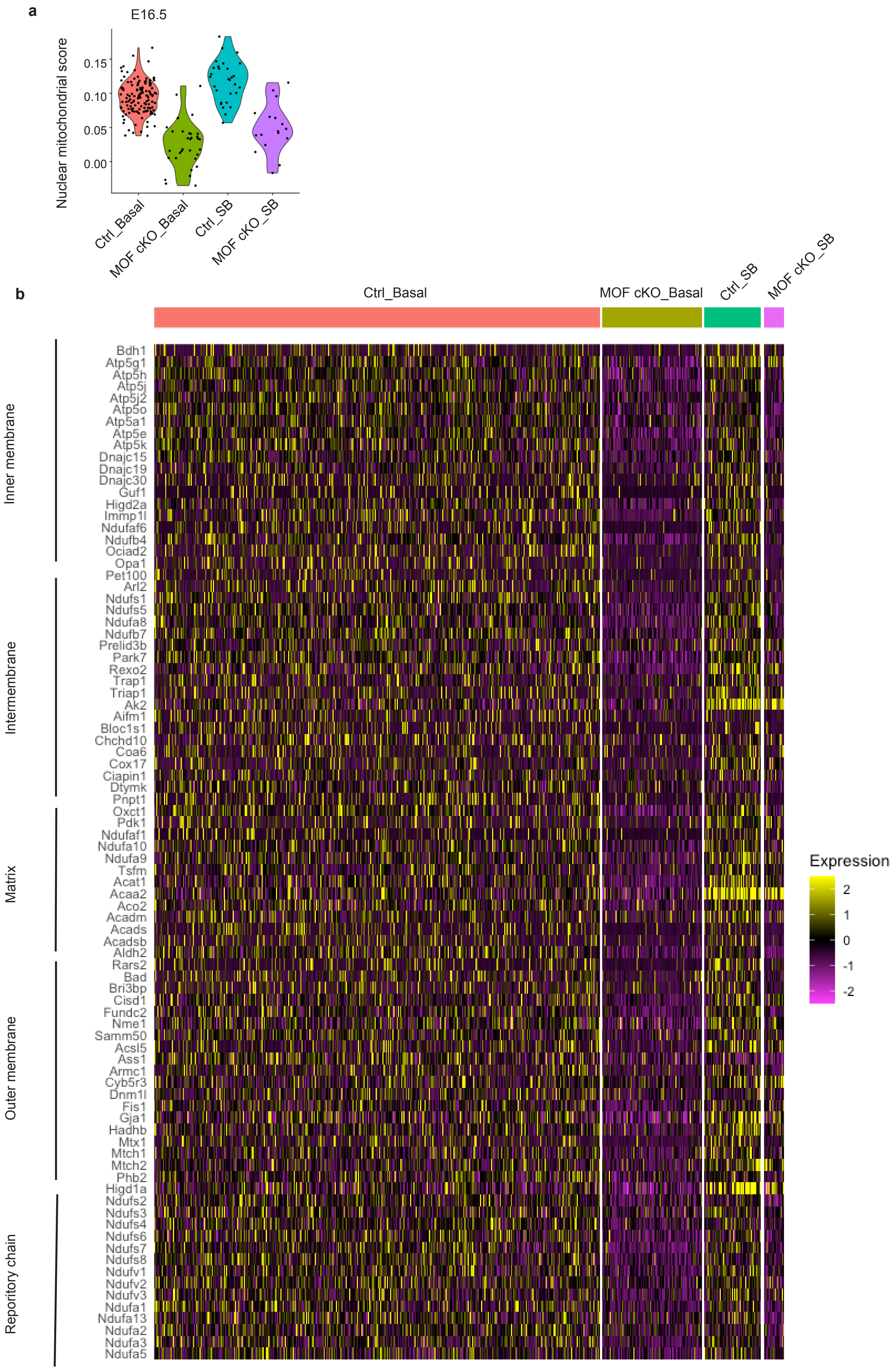
a
E16.5
Nuclear mitochondrial score
0.15
0.10
0.05
0.00
Ctrl_Basal
MOF cKO_Basal
Ctrl_SB
MOF cKO_SB
b
Ctrl_Basal
MOF cKO_Basal
Ctrl_SB
MOF cKO_SB
Inner membrane
Intermembrane
Matrix
Outer membrane
Reporitory chain
Bdh1
Atp5g1
Atp5h
Atp5j
Atp5j2
Atp5o
Atp5a1
Atp5e
Atp5k
Dnajc15
Dnajc19
Dnajc30
Guf1
Higd2a
Immp1l
Ndufaf6
Ndufb4
Ociad2
Opa1
Pet100
Arl2
Ndufs1
Ndufs5
Ndufa8
Ndufb7
Prelid3b
Park7
Rexo2
Trap1
Triap1
Ak2
Aifm1
Bloc1s1
Chchd10
Coa6
Cox17
Ciapin1
Dtymk
Pnpt1
Oxct1
Pdk1
Ndufaf1
Ndufa10
Ndufa9
Tsfm
Acat1
Acaa2
Aco2
Acadm
Acads
Acadsb
Aldh2
Rars2
Bad
Bri3bp
Cisd1
Fundc2
Nme1
Samm50
Acsl5
Ass1
Armc1
Cyb5r3
Dnm1l
Fis1
Gja1
Hadhb
Mtx1
Mtch1
Mtch2
Phb2
Higd1a
Ndufs2
Ndufs3
Ndufs4
Ndufs6
Ndufs7
Ndufs8
Ndufv1
Ndufv2
Ndufv3
Ndufa1
Ndufa13
Ndufa2
Ndufa3
Ndufa5
Expression
2
1
0
-1
-2

Supplementary Fig. 6 | Downregulated mitochondrial genes in scRNAseq. a, Aggregated nuclear-encoded mitochondrial gene expression score in E16.5 scRNAseq. **b**, Heatmap of downregulated mitochondrial genes in E15.5 scRNAseq, grouped by different mitochondrial compartments. Ctrl_Basal, control basal progenitor cells; MOF cKO_Basal, MOF cKO basal progenitor cells; Ctrl_SB, control suprabasal cells; MOF cKO_SB, MOF cKO suprabasal cells.

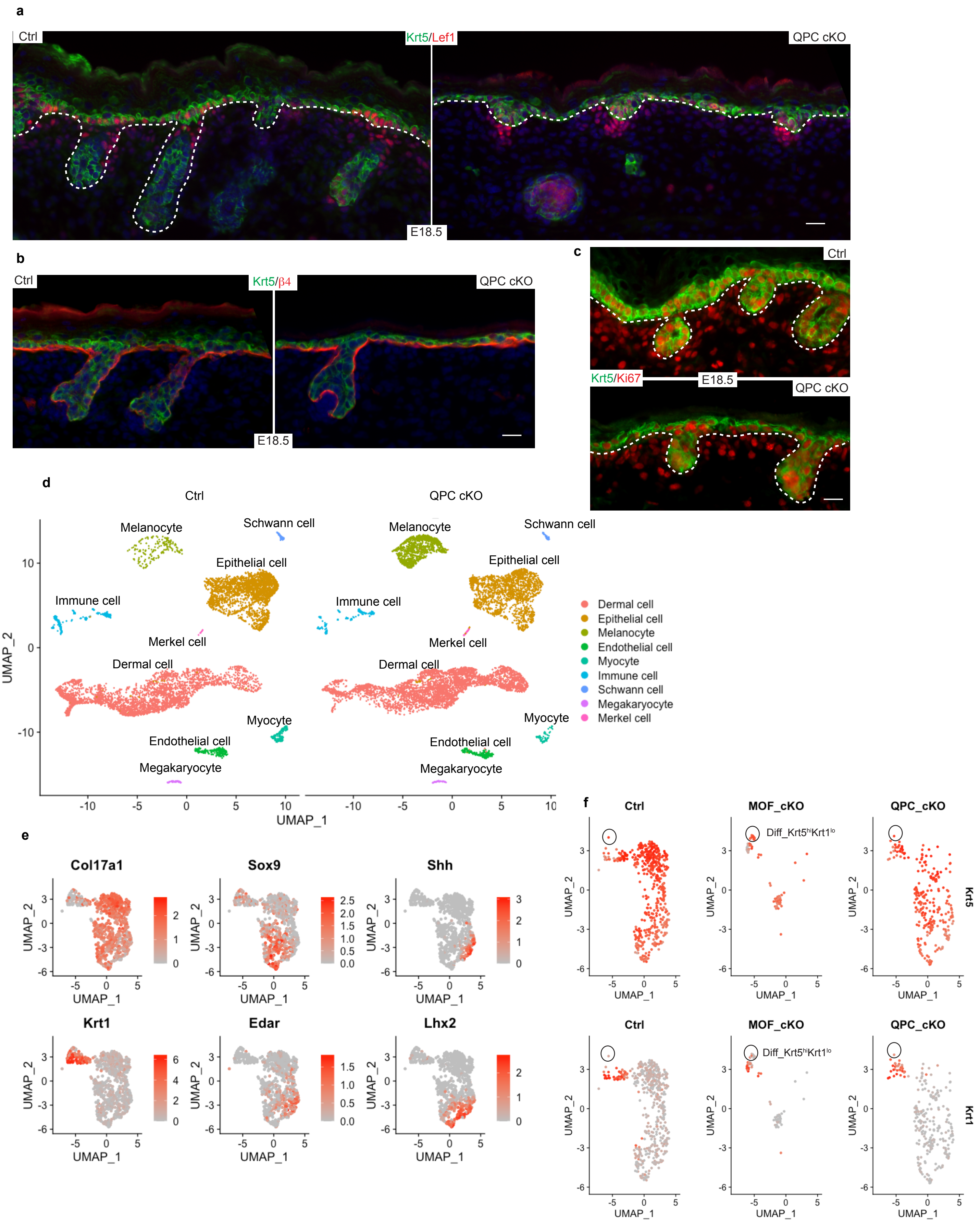
a
Ctrl
Krt5/Lef1
QPC cKO
E18.5
b
Ctrl
Krt5/β4
QPC cKO
E18.5
c
Ctrl
Krt5/Ki67
E18.5
QPC cKO
d
Ctrl
QPC cKO
Melanocyte
Schwann cell
Epithelial cell
Immune cell
Merkel cell
Dermal cell
Myocyte
Endothelial cell
Megakaryocyte
UMAP_2
UMAP_1
Dermal cell
Epithelial cell
Melanocyte
Endothelial cell
Myocyte
Immune cell
Schwann cell
Megakaryocyte
Merkel cell
e
Col17a1
Sox9
Shh
Krt1
Edar
Lhx2
f
Ctrl
MOF_cKO
QPC_cKO
Diff_Krt5hiKrt1lo
Krt5
Krt1

Supplementary Fig. 7 | QPC cKO phenotypical analysis. a, Reduced advanced stage hair follicles in E18.5 QPC cKO as shown by Lef1 staining. Representative images from three pairs of samples. **b**, Intact basement membrane as indicated by β4 integrin staining in QPC cKO. Representative images from three pairs of samples. **c**, Non-changed proliferation marked by similar proportion of Ki67+ cells in QPC cKO. Representative images from three pairs of samples. **d**, Nine cell types detected in E17.5 Ctrl and QPC cKO dorsal skin samples by scRNAseq. **e**, Feature plot of genes that were used for identifying each epithelial clusters. Col17a1 marks basal cells; Krt1 marks suprabasal cells; Sox9, Shh, Eda and Lhx2 all mark hair germ cells. **f**, Split view of Krt5 and Krt1 staining in Ctrl, MOF cKO and QPC cKO. Circle indicates Diff_Krt5hi cells, which are only detected in MOF cKO, but not in Ctrl or QPC cKO samples. Ctrl, control; Diff_Krt5^{hi}Krt1^{lo}, Krt5^{high}Krt1^{low} suprabasal cells as shown in Figure **3e**. Scale bar, 20 μm (**a**-**c**).

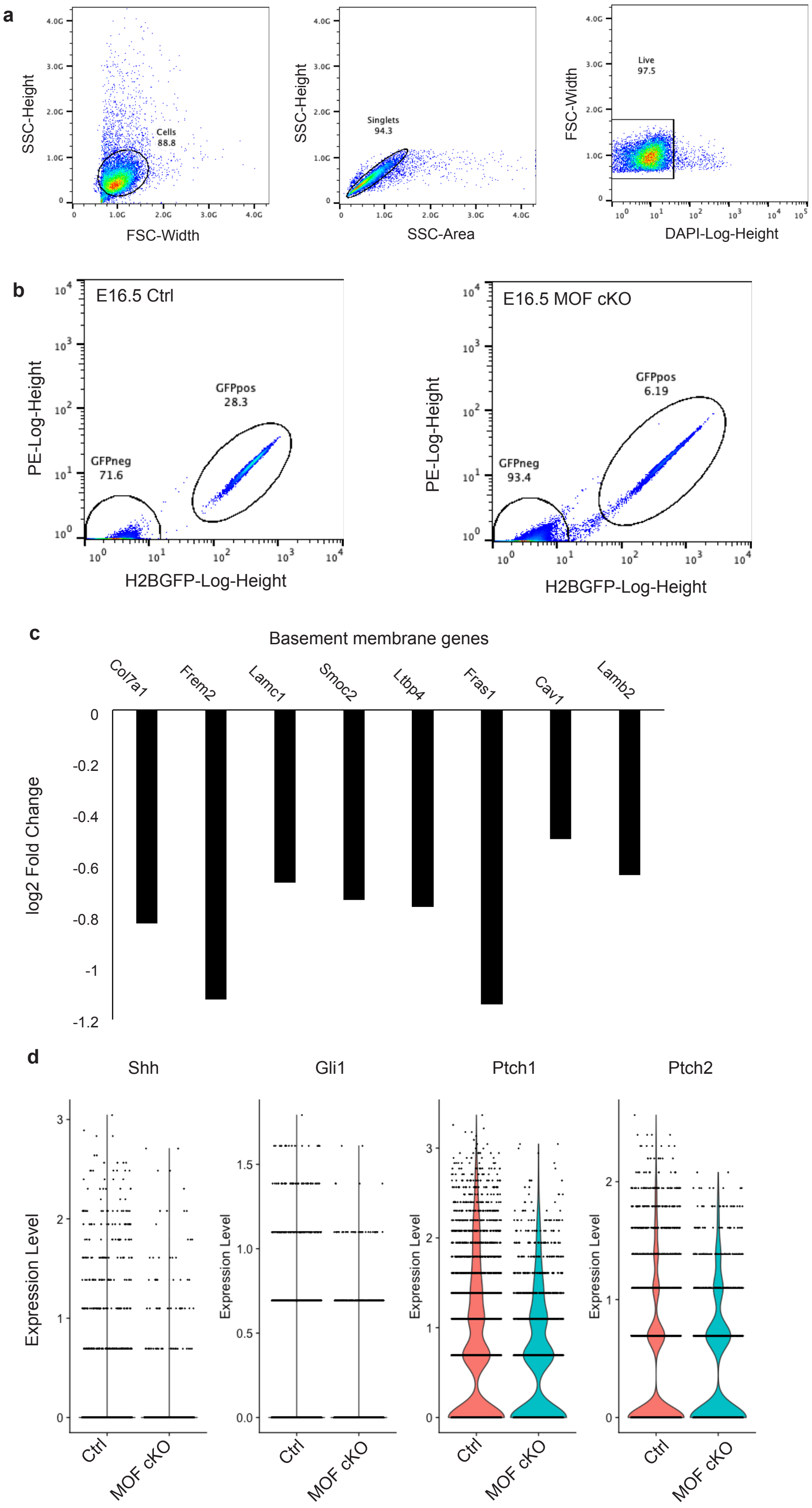

Supplementary Fig. 8 | RNAseq analysis. a, Gating strategy for flow cytometry sorting of K14-H2BGFP⁺ epithelial cells. **b**, K14-H2BGFP⁺ epithelial cell sorting profile for bulk RNAseq. c, Log2 fold change of downregulated basement membrane genes detected by bulk RNAseq. d, Downregulation of Shh and its target genes detected in E15.5 scRNAseq.

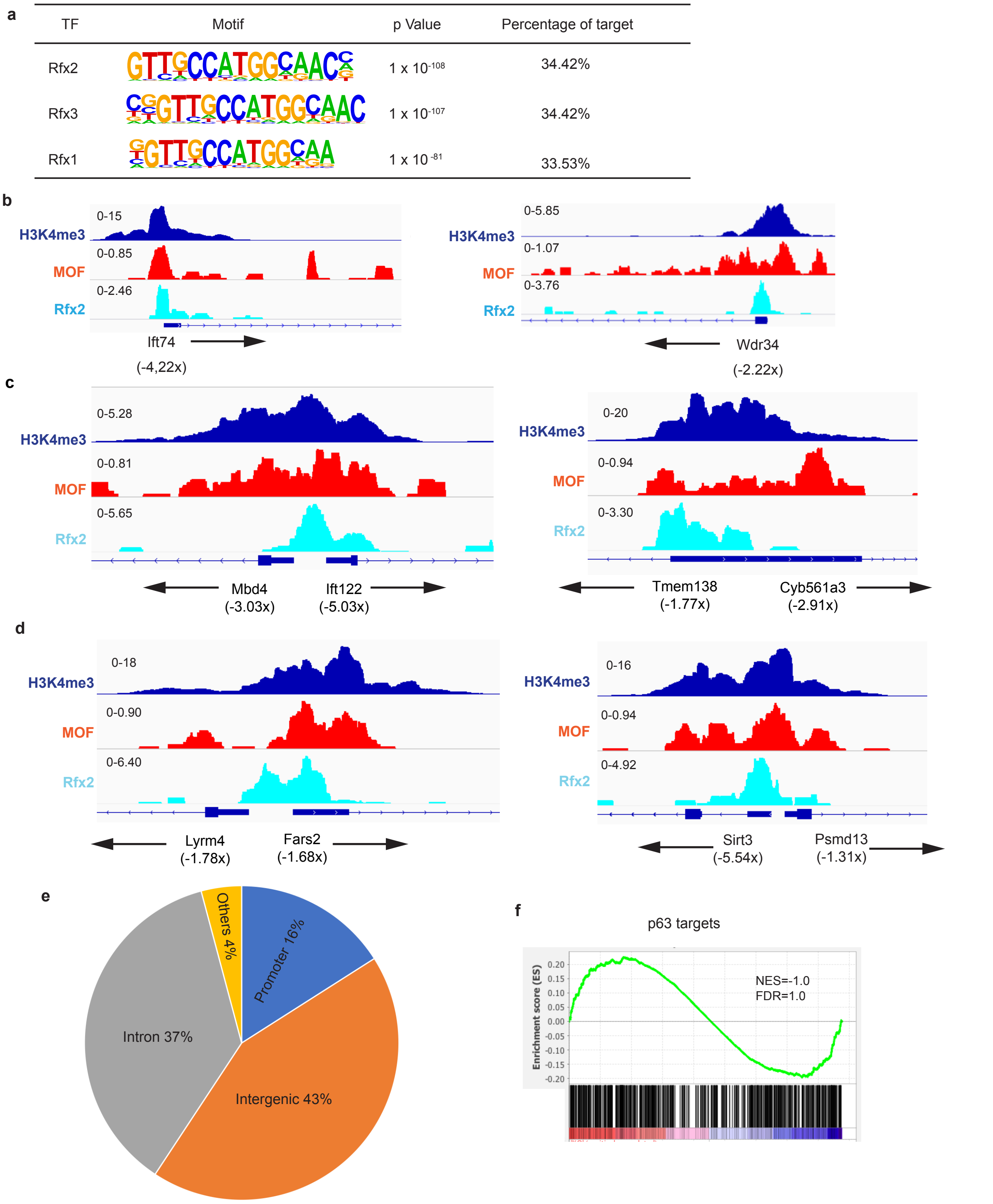

Supplementary Fig. 9 | ChIPseq analysis. a, Top enriched motifs in Rfx2 Cut&Run peaks. **b**, Examples of two ciliary genes, Ift174 and Wdr34, that have both MOF and Rfx2 binding on their promoters marked by H3K4me3. **c**, Examples of two ciliary genes, Ift122 and Tmem138, that share promoter with another gene, have MOF and Rfx2 binding on their promoters, and both genes are downregulated. **d**, Examples of two mitochondrial genes Lyrm4 and Fars2 that share one promoter, another mitochondrial gene Sirt3 shares the same promoter as Psmd13. Both shared promoters have MOF and Rfx2 binding and all four genes are downregulated in MOF cKO. **e**, Distribution of P63 Cut&Run peaks in the genome. **f**, GSEA for P63 targets identified in a previous study[27]. Values in parentheses indicate gene expression fold change calculated from bulk RNAseq. Arrows indicate the direction of gene transcription.

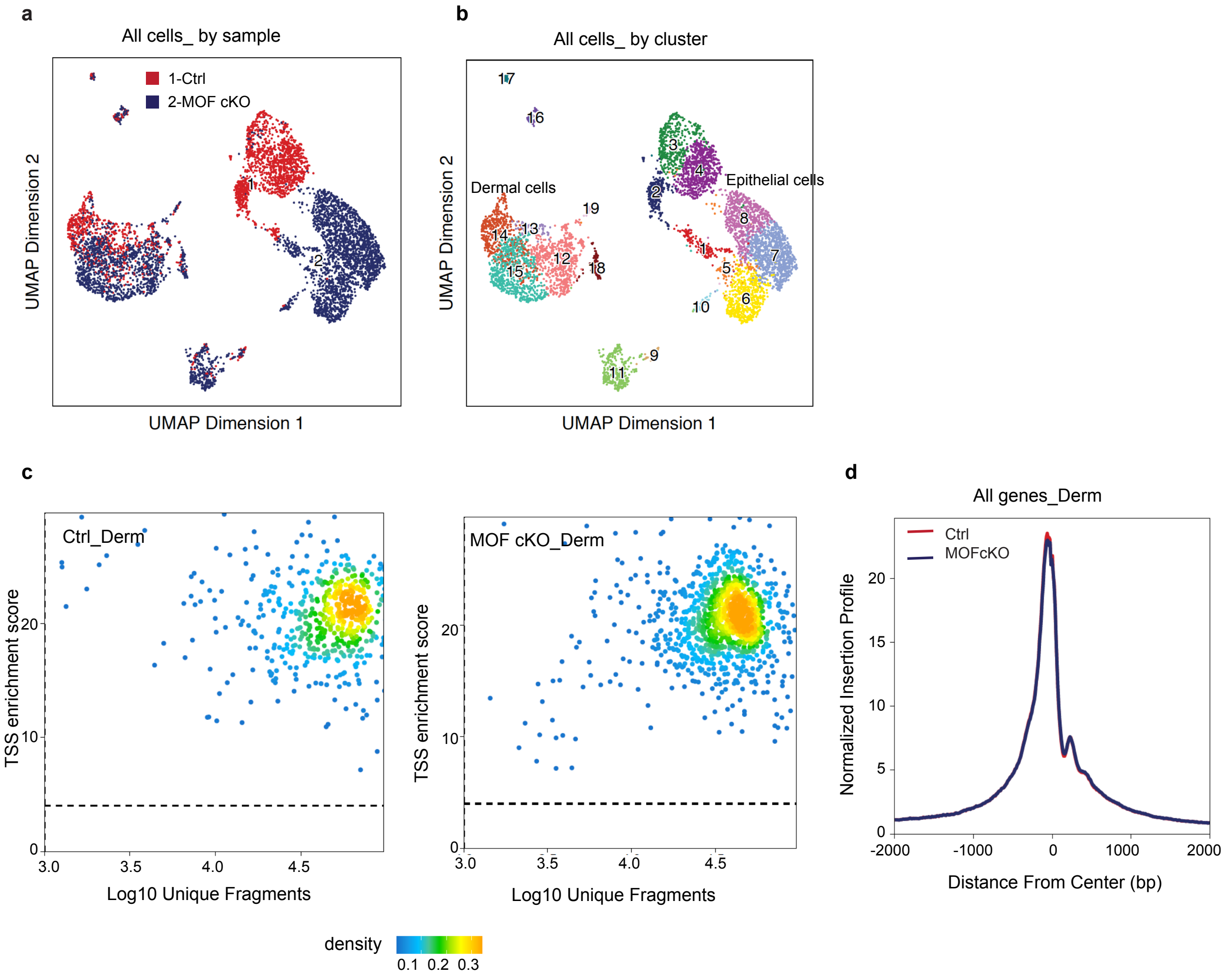

Supplementary Fig. 10 | scATACseq analysis. **a** and **b**, UMAP clustering of E16.5 scATACseq data for control (Ctrl) and MOF cKO, colored either by sample (**a**) or by cluster (**b**). **c**, Transcription start site (TSS) enrichment score *vs.* number of unique fragments calculated from scATACseq data for dermal population. Ctrl_Derm, control_dermal cells; MOF cKO_Derm, MOF cKO_dermal cells. **d**, Normalized Tn5 insertion profile for all genes in dermal cells (All genes_Derm) calculated from scATACseq data.
