# Supplementary material for: MOF-mediated histone H4 Lysine 16 acetylation governs mitochondrial and ciliary functions by controlling gene promoters
Source: Nat Commun. 2023 Jul 21;14:4404. doi: 10.1038/s41467-023-40108-0 (PMC10362062; doi:10.1038/s41467-023-40108-0)
Supplement: Supplementary file 3 — Description of Additional Supplementary Files [file 41467_2023_40108_MOESM3_ESM.pdf]

Description of Additional Supplementary Files

File Name: Supplementary Data 1
Description: Summary of number and percentage of epithelial cells in E15.5 and E16.5 scRNAseq.

File Name: Supplementary Data 2
Description: Control and MOF cKO shared basal marker genes identified in E16.5 scRNAseq.

File Name: Supplementary Data 3
Description: Epithelial lineage genes identified in E16.5 control scRNAseq by comparing epithelial cells with all other non-epithelial cells.

File Name: Supplementary Data 4
Description: The exact p value for all the quantifications.

File Name: Supplementary Data 5
Description: qPCR primer sequences.
