# Supplementary material for: MOF-mediated histone H4 Lysine 16 acetylation governs mitochondrial and ciliary functions by controlling gene promoters
Source: Nat Commun. 2023 Jul 21;14:4404. doi: 10.1038/s41467-023-40108-0 (PMC10362062; doi:10.1038/s41467-023-40108-0)
Supplement: Supplementary file 9 — Reporting Summary [file 41467_2023_40108_MOESM9_ESM.pdf]

nature portfolio

Corresponding author(s): Rui Yi

Last updated by author(s): Jun 16, 2023

Reporting Summary

Nature Portfolio wishes to improve the reproducibility of the work that we publish. This form provides structure for consistency and transparency in reporting. For further information on Nature Portfolio policies, see our Editorial Policies and the Editorial Policy Checklist.

Statistics

For all statistical analyses, confirm that the following items are present in the figure legend, table legend, main text, or Methods section.

n/a	Confirmed	
☐	☒	The exact sample size (*n*) for each experimental group/condition, given as a discrete number and unit of measurement
☐	☒	A statement on whether measurements were taken from distinct samples or whether the same sample was measured repeatedly
☐	☒	The statistical test(s) used AND whether they are one- or two-sided<br>*Only common tests should be described solely by name; describe more complex techniques in the Methods section.*
☒	☐	A description of all covariates tested
☒	☐	A description of any assumptions or corrections, such as tests of normality and adjustment for multiple comparisons
☐	☒	A full description of the statistical parameters including central tendency (e.g. means) or other basic estimates (e.g. regression coefficient) AND variation (e.g. standard deviation) or associated estimates of uncertainty (e.g. confidence intervals)
☐	☒	For null hypothesis testing, the test statistic (e.g. *F*, *t*, *r*) with confidence intervals, effect sizes, degrees of freedom and *P* value noted<br>*Give P values as exact values whenever suitable.*
☒	☐	For Bayesian analysis, information on the choice of priors and Markov chain Monte Carlo settings
☒	☐	For hierarchical and complex designs, identification of the appropriate level for tests and full reporting of outcomes
☒	☐	Estimates of effect sizes (e.g. Cohen's *d*, Pearson's *r*), indicating how they were calculated

Our web collection on statistics for biologists contains articles on many of the points above.

Software and code

Policy information about availability of computer code

Data collection	Nikon NIS Elements, version 4.51.00; Leica LAS X suite, version 3.7 for image collection. Kansl1 cKO and control RNAseq libraries, scRNAseq libraries, scATACseq libraries and Cut&Run libraries were sequenced on Illumina NovaSeq 6000; MOF cKO and control RNAseq libraries and MOF ChIPseq library was sequenced on Illumina HiSeq 2500. Summit v5.0 for flow cytometry data collection.
Data analysis	For images, ImageJ, version 2.3.0/1.53f was used to quantify the fluorescent intensity, Pericentrin angle relative to the basement membrane, epidermal thickness, cilia length and percentage of ciliated cells. Flow cytometry data were analyzed using FlowJo, version 10.8.1. RNAseq data were aligned to the mouse genome (mm10) using HISAT2 (version 2.1.0). Expression of each gene was counted using HTSeq-count (version 0.9.1). Differential analysis was carried out using DESeq2 (version 1.38.3). ChIPseq and Cut&Run data were aligned to mm10 using Bowtie2 (version 2.2.9). Peak calling used MACS2 (version 2.1.1). K-means clustering was performed using seqMINER (version 1.3.4). Single cell RNAseq libraries were mapped to mm10 using cellranger (version 6.1.2), secondary analysis was done using R package Seurat (version 4.0). Single cell ATACseq libraries were aligned to mm10 using cellranger-atac (version 2.0.0), further downstream analysis was done using R package ArchR (version 1.0.1).

For manuscripts utilizing custom algorithms or software that are central to the research but not yet described in published literature, software must be made available to editors and reviewers. We strongly encourage code deposition in a community repository (e.g. GitHub). See the Nature Portfolio guidelines for submitting code & software for further information.

Data

Policy information about availability of data

All manuscripts must include a data availability statement. This statement should provide the following information, where applicable:

- Accession codes, unique identifiers, or web links for publicly available datasets
- A description of any restrictions on data availability
- For clinical datasets or third party data, please ensure that the statement adheres to our policy

All sequencing data were deposited to NCBI/GEO Super Series under accession number GSE214441. Mouse genome mm10 is available at http://genome.ucsc.edu/cgi-bin/hgGateway?db=mm10. Mouse cilium gene set was downloaded at https://www.gsea-msigdb.org/gsea/msigdb/mouse/geneset/GOCC_CILIUM.html. Mouse mitochondrion gene set was downloaded at https://www.gsea-msigdb.org/gsea/msigdb/mouse/geneset/GOCC_MITOCHONDRION.html. Mouse reference dataset for scRNAseq was downloaded from https://support.10xgenomics.com/single-cell-gene-expression/software/downloads/latest. Mouse reference dataset for scATACseq was downloaded from https://support.10xgenomics.com/single-cell-atac/software/downloads/latest. Source data are provided with this paper.

Human research participants

Policy information about studies involving human research participants and Sex and Gender in Research.

Reporting on sex and gender	N/A
Population characteristics	N/A
Recruitment	N/A
Ethics oversight	N/A

Note that full information on the approval of the study protocol must also be provided in the manuscript.

Field-specific reporting

Please select the one below that is the best fit for your research. If you are not sure, read the appropriate sections before making your selection.

☒ Life sciences ☐ Behavioural & social sciences ☐ Ecological, evolutionary & environmental sciences

For a reference copy of the document with all sections, see nature.com/documents/nr-reporting-summary-flat.pdf

Life sciences study design

All studies must disclose on these points even when the disclosure is negative.

Sample size	No analysis was used to predetermine sample sizes. We utilized sample sizes that are commonly used in literatures with similar topic (Rodrigues 2021, Valerio 2017) and we made every effort to avoid excessive or unnecessary use of animals.
Data exclusions	No data was excluded.
Replication	All experimental data were reliably reproduced in multiple independent experiments as indicated in the figure legends.
Randomization	Mouse genotypes were predetermined by PCR, so they were not randomized.
Blinding	The investigators were not blinded for the analysis of this study. The MOF and QPC knockout animals show clear phenotype at the time of analysis, so blinding is not possible.

Reporting for specific materials, systems and methods

We require information from authors about some types of materials, experimental systems and methods used in many studies. Here, indicate whether each material, system or method listed is relevant to your study. If you are not sure if a list item applies to your research, read the appropriate section before selecting a response.

Materials & experimental systems

n/a	Involved in the study
☐	☒ Antibodies
☐	☒ Eukaryotic cell lines
☒	☐ Palaeontology and archaeology
☐	☒ Animals and other organisms
☒	☐ Clinical data
☒	☐ Dual use research of concern

Methods

n/a	Involved in the study
☐	☒ ChIP-seq
☐	☒ Flow cytometry
☒	☐ MRI-based neuroimaging

Antibodies

Antibodies used	Primary antibodies used for fluorescent staining in this study (supplier, catalogue number, effective dilution used): H4K16ac (Sigma-Aldich, #07-329, 1:2000), Krt5 (Covance, #SIG-3475, 1:2000), Krt1 (Covance, #PRB-165P, 1:2000), Loricrin (The Rockefeller University, Gift from E. Fuchs, generated in the lab, 1:1000), β4 integrin (BD Biosciences, #553745, 1:200), Col17 (Abcam, #ab184996, 1:500), Pericentrin (Covance, #PRB-432C, 1:200), E-Cadherin (The Rockefeller University, Gift from E. Fuchs, generated in the lab, 1:200), α-Catenin (Cell Signaling Technology, #3236, 1:1000), Active-Caspase3 (R&D Systems, #AF835, 1:1000), Sox9 (Millipore, #AB5535, 1:500), Ki67 (Abcam, #ab15580, 1:500), Lef1 (Cell Signaling Technology, #2230, 1:500), P63 (Cell Signaling Technology, #4892, 1:200), BrdU (Abcam, #ab6326, 1:500), Krt6 (Covance, #PRB-169P, 1:500), H3K4me3 (Cell Signaling Technology, #9751, 1:200 for IF, 1:100 for Cut&Run), H3K27ac (Cell Signaling Technology, #8173, 1:200 for IF, 1:100 for Cut&Run), Arl13b (NeuroMab, #AB_11000053, 1:10), MOF (Bethyl, #A300-992A, 1:100), H3Keme1 (Cell Signaling Technology, #9723, 1:100), Rfx2 (Sigma-Aldrich, #HPA048969, 1:100).<br>Secondary antibodies used in this study include goat anti-chicken, Alexa Fluor 488 (Invitrogen, A-11039, 1:2000), goat anti-rabbit, Alexa Fluor 555 (Invitrogen, A-21428, 1:2000), goat anti-rat Alexa Fluor 555 (Invitrogen, A-21434, 1:2000), goat anti-rat Alexa Fluor 488 (Invitrogen, A-11006, 1:2000), goat anti-mouse IgG2a Alexa Fluor 555 (Invitrogen, A-21137, 1:2000).
Validation	Antibodies were validated in previous publications of the Yi lab (Fan 2019, Hoefert 2018, Wang 2013).

Eukaryotic cell lines

Policy information about cell lines and Sex and Gender in Research

Cell line source(s)	Mouse keratinocytes were generated from wildtype newborn animals. HEK293T cells were from ATCC (CRL-11268).
Authentication	The cell line was not authenticated.
Mycoplasma contamination	The cell line was not tested for mycoplasma.
Commonly misidentified lines (See ICLAC register)	No commonly misidentified lines was used in this study.

Animals and other research organisms

Policy information about studies involving animals; ARRIVE guidelines recommended for reporting animal research, and Sex and Gender in Research

Laboratory animals	Mus musculus. All experiments were carried out following IACUC-approved protocols and guidelines at CU Boulder and Northwestern, respectively. Mice were bred and housed according to guidelines of the IACUC in a pathogen-free facility at University of Colorado at Boulder and at Northwestern University Feinberg School of Medicine. Housing rooms have 12-hr light/dark cycles with an ambient temperature of 23 oC and 50% humidity.<br>Seven weeks old heterozygous animals with Krt14-Cre were used for breeding to generate conditional knockout. For MOF related study, all experiments were performed with E15.5 and E16.5 embryos; for Kansl1 related study, all experiments were performed with P0.5 newborns; for QPC related study, all experiments were performed with E17.5 and E18.5 embryos.<br>All the mice were C57Bl/6J and CD-1 mixed background.
Wild animals	No wild animals was used in this study.
Reporting on sex	No sex based analysis in this study.
Field-collected samples	No field-collected samples was used in this study.
Ethics oversight	The animal protocols were approved by the IACUC at University of Colorado, Boulder and Northwestern University.

Note that full information on the approval of the study protocol must also be provided in the manuscript.

ChIP-seq

Data deposition

☒ Confirm that both raw and final processed data have been deposited in a public database such as GEO.

☒ Confirm that you have deposited or provided access to graph files (e.g. BED files) for the called peaks.

Data access links<br>*May remain private before publication.*	https://www.ncbi.nlm.nih.gov/geo/query/acc.cgi?acc=GSE214441
Files in database submission	Raw data include:<br>MOF.fq.gz; H4K16Ac_1_R1.fq.gz; H4K16Ac_1_R2.fq.gz; H4K16Ac_2_R1.fq.gz; H4K16Ac_2_R2.fq.gz; H4K16Ac_3_R1.fq.gz; H4K16Ac_3_R2.fq.gz; H3K4me1_R1.fq.gz; H3K4me1_R2.fq.gz; H3K4me3_R1.fq.gz; H3K4me3_R2.fq.gz; H3K4me3_rep_R1.fq.gz; H3K4me3_rep_R2.fq.gz; H3K27Ac_R1.fq.gz; H3K27Ac_R2.fq.gz; H3K27Ac_rep_R1.fq.gz; H3K27Ac_rep_R2.fq.gz; Rfx2_R1.fq.gz; Rfx2_R2.fq.gz.<br>Processed data include: MOF_peaks.bed; Rfx2_peaks.bed; H3K4me3_peaks.bed; H3K27Ac_rep_peaks.bed.
Genome browser session<br>(e.g. UCSC)	NA

Methodology

Replicates	One replicate for MOF, H3K4me1 and Rfx2, three replicates for H4K16ac and two replicates for H3K4me3 and H3K27Ac.
Sequencing depth	Sample; raw reads; mapped reads; length of reads; type of reads<br>MOF; 40,747,419; 20,106870; 151; SE<br>H4K16Ac_1; 9,961,274; 9,946,360; 150; PE<br>H4K16Ac_2; 6,310,355; 6,301,210; 150; PE<br>H4K16Ac_3; 12,739,961; 12,722,092; 150; PE<br>H3K4me1; 26,371,012; 26,231,245; 150; PE<br>H3K4me3; 15,307,836; 15,000,148; 150; PE<br>H3K4me3_rep; 45,125,370; 44,212,819, 150; PE<br>H3K27Ac; 11,725,141; 11,716,419; 150; PE<br>H3K17Ac_rep; 62;035;582; 60,174,514; 150; PE<br>Rfx2; 6,908,691; 6,904,021; 150; PE
Antibodies	MOF (Bethyl, #A300-992A, 1:100), H4K16ac (Sigma-Aldich, #07-329, 1:100), Rfx2 (Sigma-Aldrich, #HPA048969, 1:100), H3K4me3 (Cell Signaling Technology, #9751, 1:100), H3K4me1 (Cell Signaling Technology, #9723, 1:100), H3K27ac (Cell Signaling Technology, #8173, 1:100).
Peak calling parameters	MOF peaks were called using MACS2 with parameter: --keep-dup auto --nomodel<br>Rfx2, H3K4me3 and H3K27Ac peaks were called using MACS2 with parameter: BAMPE
Data quality	Data quality for all samples were visually examined in igv for robust peak. 11239 peaks were called for MOF; 337 peaks were called for Rfx2.
Software	Raw reads were mapped using Bowtie2; peaks were called using MACS2; K-means clustering was performed using seqMINER; enriched motifs were searched using Homer; data were visualized using Igv.

Flow Cytometry

Plots

Confirm that:

☒ The axis labels state the marker and fluorochrome used (e.g. CD4-FITC).

☒ The axis scales are clearly visible. Include numbers along axes only for bottom left plot of group (a 'group' is an analysis of identical markers).

☒ All plots are contour plots with outliers or pseudocolor plots.

☒ A numerical value for number of cells or percentage (with statistics) is provided.

Methodology

Sample preparation	For E16.5 sorting, total dorsal skin was mined into small pieces and incubated with 0.1% collagenase (Worthington, LS004188) for 30 min at 37oC. After incubation, PBS was used to dilute the collagenase, then pipet to dissociate. Tissues were pelleted by centrifuge and then subject to fresh Trypsin digestion for 5 min at 37oC. PBS supplemented with 5% chelated FBS was used to neutralize Trypsin and cells were filtered through 40-um cell strainer. DAPI-positive dead cells were excluded and epithelial cells were enriched by selecting Krt14-H2BGFP+ cells.
Instrument	MoFlo XDP (Beckman Coulter)

Software	Summit v5.0
Cell population abundance	Cells were sorted using the "Purity" option. Purity of sorted cells was checked by postsort and with > 90% of sorted cells falling into the gate of interest.
Gating strategy	Main cell population was gated based on cell size (FSC) and complexity (SSC), singlets were gated based on SSC width, live cells were gated as DAPI-negative, epithelial cells were gated as K14-H2BGFP-positive. All gating has a clear negative and positive separation. A figure exemplifying the gating strategy is provided in Extended Data Figure 8a.

☒ Tick this box to confirm that a figure exemplifying the gating strategy is provided in the Supplementary Information.
